# Dissipation of Four Typical Insecticides on Strawberries and Effects of Different Household Washing Methods

**DOI:** 10.3390/foods12061248

**Published:** 2023-03-15

**Authors:** Wenting Wang, Jong-Wook Song, Seong-Hoon Jeong, Jung-Hoon Jung, Jong-Su Seo, Jong-Hwan Kim

**Affiliations:** Environmental Safety-Assessment Center, Korea Institute of Toxicology (KIT), Jinju 52834, Republic of Korea

**Keywords:** pesticides, metabolites, washing methods, removal, risk assessment

## Abstract

The dissipation patterns of chlorfenapyr, cyenopyrafen, indoxacarb, and spirotetramat on strawberries and the effects of different household washing methods were investigated. A risk assessment was also conducted by monitoring the insecticide residues detected. The concentrations ranged from 0.011 to 0.27 mg/kg for chlorfenapyr, 0.064 to 0.99 mg/kg for cyenopyrafen, 0.042 to 0.53 mg/kg for indoxacarb, and from 0.25 to 1.3 mg/kg for spirotetramat, which were all below the maximum residue limits (MRLs) reported. Soaking the fruit in solution and then rinsing with running water (B) led to better residue removal (40.9 ± 23.7%) than only soaking in solution (A) (24.7 ± 22.5%). However, neither method decreased chlorfenapyr concentrations, suggesting that the physical–chemical properties of chlorfenapyr could also affect its removal on strawberries. Regarding the different washing solutions in method B, 3% vinegar (removal efficiency: 48.7%) and 3% salt (45.7%) were the most efficient, followed by 3% green tea (38.9%), and tap water only (24.6%). Additionally, the estimated risk quotients (RQs) for strawberry consumption for women were about 1.5 times higher than those observed for men, but both were lower than 1, suggesting minimal risk to humans.

## 1. Introduction

Strawberries are widely consumed, and their health benefits are well-known [1,2]. To increase production, strawberries are cultivated in greenhouses under high temperatures and humid conditions, which are favorable for the occurrence of pests and pathogens [3,4]. Various insecticides, such as chlorfenapyr and cyenopyrafen, are mainly used commercially for crop protection against a range of insect and mite pests [5]. Crops, turfgrasses, and ornamental landscape plants can be treated with indoxacarb to control lepidopteran insects. Spirotetramat is an insecticide derived from tetramic acid, a systemic material used to control insects in their juvenile and immature stages, including aphids, scale insects, and whiteflies [6]. However, insecticides are applied multiple times during the growth period of strawberries [7], and it is difficult to guarantee the complete elimination of insecticide residues in strawberries. Moreover, humans can be exposed to insecticides directly through food consumption, which increases potential food safety risks. Thus, in South Korea, the maximum residue limits (MRLs) in strawberries are regulated, set by the Ministry of Food and Drug Safety (MFDS), and vary by insecticide: for chlorfenapyr, it is 0.5 mg/kg, for cyenopyrafen, 2.0 mg/kg, for indoxacarb, 1.0 mg/kg, and for spirotetramat, 3.0 mg/kg. Meanwhile, in the European Union (EU) and the United States (US), different MRL values were also established for chlorfenapyr (EU: 0.05 mg/kg; US: 2.0 mg/kg), cyenopyrafen (0.02 mg/kg; 0.6 mg/kg), indoxacarb (0.1 mg/kg; 0.3 mg/kg), and spirotetramat (0.05 mg/kg; 3.0 mg/kg).

Meanwhile, in fruit, most insecticide residues are retained on peel surfaces. Researchers found that peeling, washing, and soaking fruit in solutions with chemicals such as chlorine, chlorine dioxide, hydrogen peroxide, ozone, acetic acid, or hydroxy peracetic acid is highly effective (36–100%) at reducing pesticide residues in food [8]. However, applying these chemical washing methods to household washing is inconvenient. Additionally, other household processing methods, including washing with tap water, salt solution, lemon water, baking soda, or vinegar, were performed to evaluate the resulting decreases in pesticide residues in food [9,10,11,12,13,14]. However, the results obtained were not entirely consistent. Harinathareddy et al. (2015), Raveendranath et al. (2014), and Soliman (2001) reported that the salt solution was the best risk mitigation method, whereas Acoglu et al. (2021) and Yang et al. (2017) reported that a baking soda solution can reduce pesticides more effectively from apple and orange surfaces. Polat et al. (2019) found that lemon water washing was more effective than the other methods. For the four mentioned insecticides, previous studies also reported that washing and peeling okra fruit, sweet persimmons, eggplants, and strawberries can efficiently decrease their residue levels [15,16,17,18]. Conversely, the same residues are concentrated by juicing and cooking [17]. However, to date, limited information has been obtained on removing these four insecticides from strawberries by using different household washing methods.

Thus, the dissipation patterns and impact of various household washing methods on the four primary insecticides found in strawberries were studied. The acceptable daily intake (ADI) serves as the criterion for risk assessment of insecticide residues in food consumption, accounting for the chronic toxicity of insecticides to humans [19]. The insecticide residue detected in strawberry samples was also used to assess risk.

## 2. Materials and Methods

### 2.1. Reagents and Materials

Analytical grade cyenopyrafen (99.8% purity) was purchased from Fujifilm Wako (Osaka, Japan), and chlorfenapyr (99.2%), indoxacarb (96.1%), spirotetramat (98.7%), and its four metabolites (BYI08330-cis-keto-hydroxy, BYI08330-mono-hydroxy, BYI08330-enol-glucoside, and BYI08330-cis-enol) (>97.9%) were obtained from Sigma-Aldrich (Burlington, MA, USA). HPLC-grade methanol, water, and acetonitrile (Burdick & Jackson, Muskegon, MI, USA) were used. The LC mobile phase was also prepared with HPLC-grade formic acid (>98%) and ammonium formate (>98%), both of which were procured from Sigma-Aldrich (Burlington, MA, USA). QuEChERS extraction packets and d-SPE tubes were received from Phenomenex (Torrance, CA, USA). A Z383K (HERMLE Labortechnik GmbH, Wehingen, Germany) refrigerated centrifuge and a CK2000 (Thmorgan, Beijing, China) shaker were used.

### 2.2. Field Trials and Sampling Campaign

The study was carried out at three field trial sites (IDs: SC-20-5; 34°59′38.5″N 128°02′52.0″E, GH-20-6; 35°20′44.3″N 128°48′02.7″E, and GC-20-7; 35°43′00.6″N 128°00′07.5″E) that were separated by distances of more than 80 km and were located in strawberry-growing regions of South Korea (Figure S1). Strawberry plants (variety Maehyang) were cultivated in a greenhouse. Each field trial comprised triplicates of treated and untreated (control) plots that were cultivated in the same manner, and the area of each plot was greater than 20 m^2^. Buffer zones of 2–3 m^2^ between replicate plots or between treated and untreated plots were also maintained to avoid contamination. Before commencing the trial, an assessment was conducted on all trial sites to verify that the target compound had not been previously applied or used. In addition, apart from the application for the present study, no formulated products containing the target compounds were sprayed during the trial period. Each solution of test insecticide was diluted 1000- or 2000-fold with water and sprayed twice (chlorfenapyr) or thrice (cyenopyrafen, indoxacarb, and spirotetramat) using a CO_2_-pressurized backpack boom sprayer (Model T, Bellspray, Inc., Opelousas, LA, USA) with a 7-day interval in accordance with the Good Agriculture Practice (GAP) provided by the Korea Crop Protection Association (KCPA) in South Korea. Detailed information on the spraying of the four insecticides is presented in Table S1. The average temperature and humidity were 18.4 °C and 64.7% at SC-20-5, 17.7 °C and 78.9% at GH-20-6, and 20.4 °C and 62.8% at GC-20-7 during the test period.

Except for the spirotetramat samples, the samples were collected on days 0 (3 h after insecticide application), 1, 2, 5, 7, and 14 after the last insecticide application from points across the entire plot, excluding the plot edges and ends of the rows. The spirotetramat samples were harvested on days 0 (3 h after insecticide application), 1, 3, 5, 7, and 14, considering the PHI value established for spirotetramat in South Korea. In order to prevent cross-contamination, samples were collected from untreated plots prior to collecting samples from treated plots. Samples weighing over 1 kg were collected, labeled, and placed in plastic containers. They were then transported to the laboratory at a temperature of 2–8 °C. Before setting up the MRLs of insecticides for strawberries, different strawberry forms were used for the pretreatment. So far, OECD and CODEX have used strawberries excluding caps (also called the calyx) for pretreatment, whereas Taiwan has used whole strawberries, including caps, for pretreatment [20,21,22]. Thus, to compare the residue and removal differences of the target insecticides, all samples were weighed and homogenized with or without the removal of the strawberry caps. Afterward, each sample was weighed to 10 g and kept at a temperature lower than −20 °C until analysis.

### 2.3. Household Washing Methods

To find a strawberry washing method that can effectively remove insecticides at home, we carried out the following washing steps to compare the removal of target insecticides from strawberries, which were modified from those of a previous study [23]. Based on the pre-harvest interval (PHI) set in Korea, samples collected on day 2 for chlorfenapyr, cyenopyrafen, and indoxacarb (PHI = day 2) and on day 3 for spirotetramat (PHI = day 3) after the last insecticide application were used in the present study.

#### 2.3.1. Washing with Soaking Solution

A total of 500 mL of 3% of green tea, salt, or vinegar solution was prepared in each aluminum tray. The solutions were prepared by mixing 15 g of green tea powder (S1) or salt (S2) in 500 mL of tap water and 250 mL of 6% vinegar mixed with 250 mL of tap water (S3). Additionally, 500 mL of tap water only (S4) was used as a commonly used household strawberry washing solution. The fruit was soaked in these four easily prepared household washing solutions for about 5 min. They were stirred 2–3 times.

#### 2.3.2. Washing with a Soaking Solution and Running Water

Another washing method entailed combining a soak in each solution with running water. After soaking the fruit in any of the four washing solutions for about 5 min, washing with tap water for 1 min was performed for each method.

### 2.4. Sample Pretreatment and Analysis

Each homogenized sample (10 g), both with and without caps, underwent extraction by being mixed with 10 mL of acetonitrile and shaken for 10 s. Then, 4 g of magnesium sulfate, 1 g of sodium chloride, 1 g of sodium citrate tribasic dihydrate, and 0.5 g of sodium citrate dibasic sesquihydrate were added to the mixture and agitated for 3 min using an automated shaker (1000 rpm). Following this, the samples were subjected to centrifugation at 4500 rpm (4 °C) for 5 min. Then, 7 mL of the supernatant was cleaned using 900 mg magnesium sulfate, 150 mg primary secondary amine, and 150 mg end-capped C18 sorbent with vigorous manual shaking for 10 s. After shaking, the samples were centrifuged at 4500 rpm (4 °C) for 3 min. Afterward, the resulting supernatants were filtered with a 0.22-µm syringe filter. The obtained filtrates were then diluted 10 times with acetonitrile and subjected to analysis via liquid chromatography-tandem mass spectrometry (LC–MS/MS, 6420 Triple Quad, Agilent Technology, USA). On the other hand, chlorfenapyr was analyzed using gas chromatography–tandem mass spectrometry (GC-MS/MS, SCION Triple quadrupole, Bruker, Billerica, MA, USA). LC-MS/MS in ESI ion mode and GC-MS/MS in EI ion mode were used to determine the residual target compounds in the strawberry samples. Product ions with both quantification and qualification ions were set for all target compounds. Tables S2 and S3 contain information on the LC-MS/MS and GC-MS/MS conditions.

### 2.5. Method Validation and Storage Stability

Prior to analyzing treated and untreated (control) samples harvested from the field trial sites, specificity, linearity, and recovery tests for target compounds were performed using control samples. For specificity, samples harvested on day 0 at each field trial site (untreated plots) were used as representative control samples to indicate the absence of interference. The limit of detection (LOD) and limit of quantitation (LOQ) were greater than the signal-to-noise ratios of 3:1 and 10:1, the lowest levels of the target analyte for the matrix of each trial site. Matrix-matched calibrations were used to compensate for matrix effects. Seven to eight concentrations ranging from 0.0001 to 0.2 mg/L for cyenopyrafen, chlorfenapyr, indoxacarb, and spirotetramat and its metabolites were used to assess the linearity of all steps. To conduct the recovery test, samples from untreated (control) plots at each trial site were fortified with different LOQ levels, namely 10 and 50 times the LOQ level, and performed in five replicates. Quality control (QC) samples were also prepared at 0.01 mg/kg to ensure the accuracy of the LC-MS/MS and GC-MS/MS analyses of the samples. Additionally, after sampling and sample pretreatment, storage stability samples were prepared by spiking a known amount of target insecticides into the processed untreated samples (0 day) to a final concentration of 0.5 mg/kg, and these samples were stored under the same conditions and for the same period as the treated samples.

### 2.6. Determination of Residual Concentrations

The residual concentrations of cyenopyrafen, chlorfenapyr, and indoxacarb were the only parent compounds detected in the strawberries. However, the residual definition of spirotetramat was calculated as the sum of the parent compound and its detected metabolites as shown in Equation (1):

Total spirotetramat (mg/kg) = R_S_ + (R_C_ × 1.18) + (R_M_ × 1.23) + (R_E_ × 0.81) + (R_CE_ × 1.24) (1)

where R_S_, R_C_, R_M_, R_E_, and R_CE_ are the concentrations of spirotetramat, BYI08330-cis-keto-hydroxy, BYI08330-mono-hydroxy, BYI08330-enol-glucoside, and BYI08330-cis-enol, respectively, and 1.18, 1.23, 0.81, and 1.24 are the conversion factors calculated according to the ratio of the molecular weights of the parent compound and its metabolites.

### 2.7. Half-Lives of Test Insecticides

The dissipation patterns of test insecticides in strawberries over time were evaluated using a first-order kinetic model, and the dissipation dynamics equation and half-life were calculated using Equations (2) and (3), respectively:

C_t_ = C_0_e^−kt^ (2)

t_1/2_ = ln(2)/k (3)

where C_t_ (mg/kg) represents the concentration of the residual insecticides at time t (day), C_0_ (mg/kg) represents the initial concentration of the residual insecticides at time t = 0, and k (day^−1^) represents the degradation coefficient [24].

### 2.8. Dietary Exposure Risk Assessment

To calculate the daily intake (mg/kg body weight/day) of the target insecticides in strawberries, the estimated daily intake of the four chemicals in strawberries for Korean people was calculated using Equation (4), which was developed by the U.S. Environmental Protection Agency.

Estimated daily intake (EDI) = C × R/BW(4)

This equation includes the concentrations of the four insecticides found in strawberries (C, in mg/kg), strawberry consumption rate (R, in g/d), and average body weight (BW, in kg). Table S6 displays data on the mean body weight and strawberry consumption in South Korea, which were provided by the Korea Health Industry Development Institute and the National Survey of Exposure Factors for Korean Adults and Children (KHIDI, 2019).

To assess the potential health risks associated with strawberry consumption, the estimated exposure doses (EDI) of the four insecticides were compared against the acceptable daily intake (ADI) levels recommended by the National Institute of Agricultural Sciences. The ADI values for chlorfenapyr, cyenopyrafen, indoxacarb, and spirotetramat were 0.026, 0.051, 0.010, and 0.050 mg/kg body weight/day, respectively. The chronic risk posed by these four insecticides from strawberries was calculated using the risk quotient (RQ) as shown in Equation (5):

Risk quotient (RQ) = EDI/ADI(5)

## 3. Results and Discussion

### 3.1. Method Validation and Storage Stability

**Figure 1:**
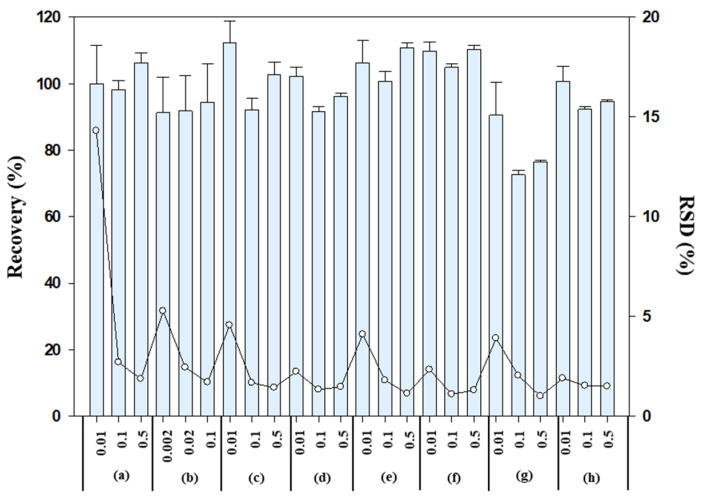
Recovery (%) and RSD (%) observed for the analyzed insecticides in strawberries: (**a**) chlorfenapyr, (**b**) cyenopyrafen, (**c**) indoxacarb, (**d**) spirotetramat, (**e**) BYI08330-cis-keto-hydroxy, (**f**) BYI08330-mono-hydroxy, (**g**) BYI08330-enol-glucoside, and (**h**) BYI08330-cis-enol.

LC-MS/MS and GC-MS/MS analyses showed that untreated samples from all trial sites had no interference peaks, and the limit of detection (LOD) and limit of quantitation (LOQ) ranged from 0.0005 to 0.005 mg/kg and from 0.002 to 0.01 mg/kg, respectively. The linearity achieved was good (R^2^ > 0.998). Moreover, the average recoveries and relative standard deviations (RSDs) of all insecticides and metabolites ranged from 72.1 to 116% with RSDs less than 14.3%, which met the criteria of the International European Commission [25] and Codex guidelines [26] (Figure 1).

To evaluate the stability of insecticide residues in strawberries, a storage stability test was conducted, as residues could degrade, decay, or dissipate even under frozen conditions below −20 °C. The results of this test are summarized in Table S4 and indicate that the analytes were considered stable if the degradation rate was less than 30%, as defined by the Joint FAO/WHO Meeting on Pesticide Residues Training Manual. The test revealed almost no degradation and demonstrated the stability of each insecticide. However, spirotetramat was shown to gradually decrease over time, which may convert to the enol during storage on the basis of the stability data of other stored analytical samples such as tomato, potato, lettuce, and almond that showed significant loss to the spirotetramat enol [26].

### 3.2. Analytical Reduction of Insecticide Residues and Half-Lives

**Figure 2:**
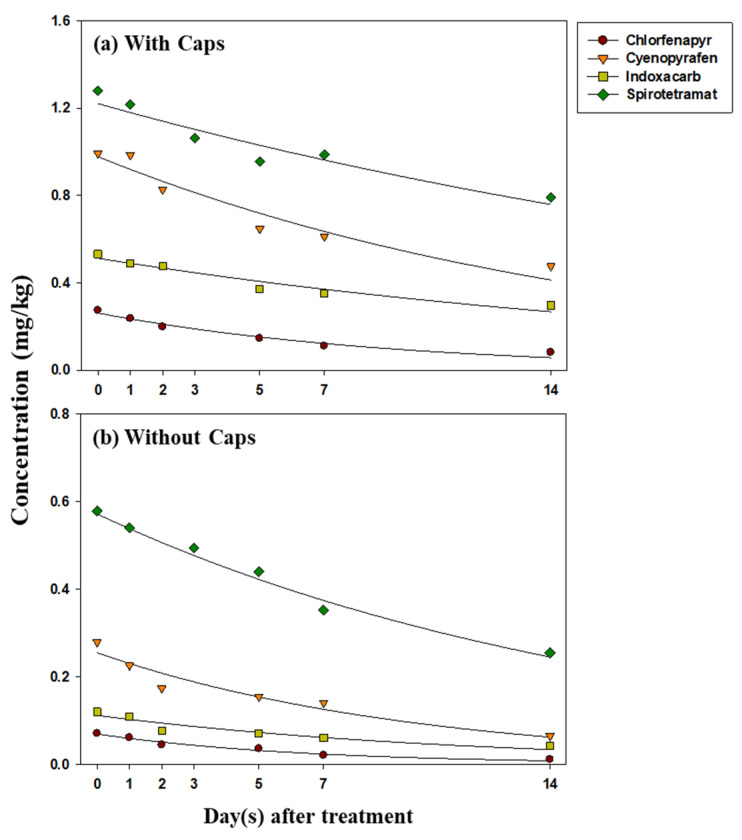
Residue decline trends of target compounds in strawberries with caps (**a**) and without caps (**b**) over 14 days.

The dissipation patterns of chlorfenapyr, cyenopyrafen, indoxacarb, and spirotetramat in strawberries are presented in Figure 2, and their half-lives were calculated using Equations (2) and (3), the results of which are shown in Table 1.

The initial depositions of chlorfenapyr, cyenopyrafen, indoxacarb, and spirotetramat in treated strawberries with caps were 0.27, 0.99, 0.53, and 1.3 mg/kg, respectively. The initial depositions of chlorfenapyr, cyenopyrafen, indoxacarb, and spirotetramat in strawberries without caps were 0.071, 0.28, 0.12, and 0.58 mg/kg, respectively. The residual amount of chlorfenapyr, cyenopyrafen, indoxacarb, and spirotetramat in strawberries with caps for day 14 were 0.081, 0.48, 0.30, and 0.79 mg/kg, respectively, and in strawberries without caps, they were 0.011, 0.064, 0.042, and 0.25 mg/kg, respectively (Table S5). The detected initial depositions of the four insecticides with or without caps were all below the MRLs for chlorfenapyr (0.5 mg/kg), cyenopyrafen (2.0 mg/kg), indoxacarb (1.0 mg/kg), and spirotetramat (3.0 mg/kg) reported in MFDS. In the case of spirotetramat, although BYI08330-mono-hydroxy was not detected in all samples, other metabolites of BYI08330-cis-enol, BYI08330-cis-keto-hydroxy, and BYI08330-enol-glucoside were only found in the samples of strawberries with caps. Of these, BYI08330-cis-enol was a major metabolite in a reaction that involved the hydrolytic cleavage of carbonate ester group of the spirotetramat, which subsequently degraded to BYI08330-cis-keto-hydroxy via further reduction of the double bond in the tetramic acid moiety and via hydroxylation. Partly, the metabolite bearing a hydroxy group was conjugated with glucuronic acid to form BYI08330-enol-glucoside [27]. 

The half-lives of chlorfenapyr, cyenopyrafen, indoxacarb, and spirotetramat in strawberries with and without caps were 4.43–6.36 days, 6.86–11.2 days, 8.04–14.9 days, and 11.4–20.4 days, respectively. The half-lives of chlorfenapyr estimated in this study were similar to or slightly lower than those reported in previous studies on strawberries (4.68 days), sweet persimmons (8.8 days), and various vegetables (1.2–9.8 days) [7,16,28,29]. The half-lives of cyenopyrafen were previously reported to be 5.2–9.8 days in Asian pears [30] and 7.99–12.65 days in grapes [31], which are similar to our current study. The half-lives of indoxacarb were reported to be 3.0–3.8 days in eggplants, 3.12–3.21 days in tomatoes, 3.46–4.77 in green chilis, 7.4–8.4 days in pomegranate fruits, and ranging from 20 to 24 days in apples [32,33,34,35,36]. The half-lives of spirotetramat were reported as 1.30–1.90 days in chilis, 5.6–7.6 days in grapes, and 4.7–9.6 days in citrus, which are shorter than those observed in this study [37,38,39,40]. These results suggest that many factors could influence the degradation of pesticides, such as their physical and chemical properties, environmental conditions, crop species, and growth dilution factors [30,41].

Table S5 also presents the average amount of residual insecticide collected on the harvest day, according to the Korean PHI (2–3 days). The residue amounts after two days of chlorfenapyr, cyenopyrafen, indoxacarb, and spirotetramat treatment in strawberries with caps were 0.20, 0.83, 0.48, and 1.1 mg/kg (after three days), respectively. The residual amounts of chlorfenapyr, cyenopyrafen, indoxacarb, and spirotetramat in strawberries without caps were 0.044, 0.17, 0.077, and 0.49 mg/kg, respectively. The final chlorfenapyr, cyenopyrafen, indoxacarb, and spirotetramat residues observed in strawberries with and without caps were all within the established MRLs (chlorfenapyr: 0.5 mg/kg, cyenopyrafen: 2.0 mg/kg, indoxacarb: 1.0 mg/kg, and spirotetramat: 3.0 mg/kg) in MFDS.

**Figure 3:**
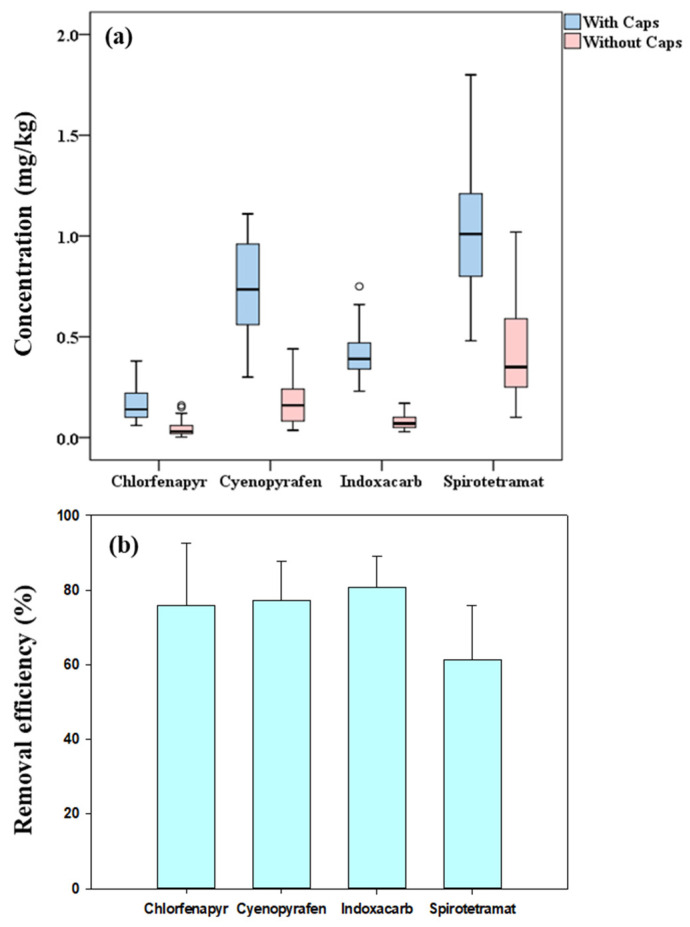
Residue concentrations (**a**) and removal efficiencies (**b**) of four insecticides in strawberries after removing caps.

The residual concentrations of the four insecticides detected in strawberries with and without caps collected from three field trials in South Korea are shown in Figure 3a. As shown, statistical significantly higher levels of chlorfenapyr (0.17 ± 0.090 mg/kg), cyenopyrafen (0.76 ± 0.22 mg/kg), indoxacarb (0.42 ± 0.11 mg/kg), and spirotetramat (1.1 ± 0.34 mg/kg) were detected in strawberries with caps than those observed in strawberries without caps (chlorfenapyr: 0.041 ± 0.040 mg/kg; cyenopyrafen: 0.17 ± 0.10 mg/kg; indoxacarb: 0.082 ± 0.041 mg/kg; spirotetramat: 0.44 ± 0.28 mg/kg) (Mann–Whitney U test, *p* < 0.01). Moreover, as shown in Figure 3b, the analyzed insecticides decreased (61.3–80.8%) in strawberries without caps, suggesting that these insecticides have more residue in the caps of strawberries than in the fruits.

### 3.3. Dissipation Pattern Removal Efficiency of Different Washing Methods

**Figure 4:**
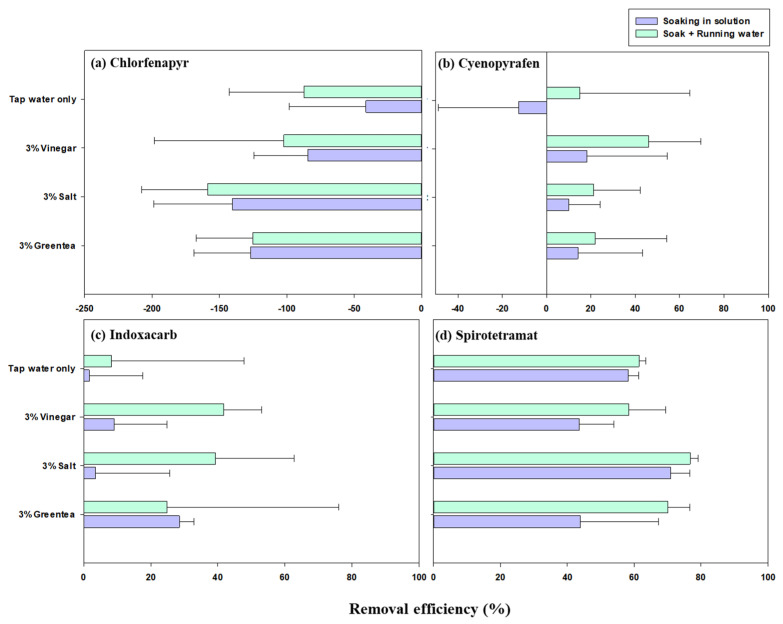
Removal efficiencies of chlorfenapyr (**a**), cyenopyrafen (**b**), indoxacarb (**c**), and spirotetramat (**d**) for strawberries using different washing methods.

The results of the removal of the four insecticides using different household washing solutions and processing treatments on strawberries (PHI = 2–3 days) are shown in Figure 4. The results reveal that the combination of the method involving soaking the strawberries in solution with the running water method (B) more efficiently removed cyenopyrafen (27.0 ± 28.4%), indoxacarb (28.5 ± 32.9%), and spirotetramat (67.1 ± 9.70%) relative to soaking in solution alone (A) (cyenopyrafen: 9.24 ± 33.8%; indoxacarb: 10.7 ± 17.5%; spirotetramat: 54.1 ± 16.3%).

However, both methods produced negative removal efficiencies for chlorfenapyr, as the removal efficiencies ranged from −140% to −41.4% for method A and from −159% to −87.3% for method B. This suggests that the chlorfenapyr residues remaining on the strawberries were not removed or even increased when using either method. Even though few studies have reported on this phenomenon so far, it can be said that when strawberries are washed in water, some of the pesticide residue on the cap may be removed and can potentially transfer to the fruit surface. Meanwhile, the relatively low water solubility (0.11 mg/L at 20 °C), systemic activity, and stability of chlorfenapyr could also be the factors which make it difficult to remove from strawberries through simple washing or rinsing. Moreover, removal efficiencies more than three times greater were estimated for spirotetramat in both methods (60.3 ± 14.8%) than for chlorfenapyr (−108 ± 60.0%), cyenopyrafen (18.5 ± 31.6%), and indoxacarb (19.6 ± 27.3%). This can also be explained by the relatively high water-solubility of spirotetramat, which resulted in the re-dissolution of this compound in water during the household washing process and its subsequent disposal through the flowing water.

Additionally, four easy homemade soaking solutions were prepared and used to compare their removal efficiencies of the analyzed insecticides. As shown in Figure 4, in method B (green bar), both positive and relatively high removal efficiencies (>40%) for three insecticides, all except chlorfenapyr, were observed for 3% vinegar (48.7 ± 16.0%) and 3% salt (45.7 ± 29.2%), followed by 3% green tea (38.9 ± 38.3%) and tap water only (24.6 ± 36.5%). In method A (purple bar), more than 20% removal efficiencies for the three insecticides, all except chlorfenapyr, were observed for 3% salt (30.4 ± 36.2%), 3% green tea (28.9 ± 22.8%), and 3% vinegar (23.6 ± 25.6%). Relatively low removal efficiencies were observed for tap water only (19.7 ± 39.6%), which is consistent with method B and other previous studies [10,42]. However, besides the negative removal efficiencies estimated for chlorfenapyr, not washing the fruit after removing the strawberry caps resulted in relatively low residues (0.019–0.032 mg/kg) of this compound compared to the use of methods A (0.033–0.081 mg/kg) and B (0.042–0.079 mg/kg). This suggests that an efficient way to remove chlorfenapyr from strawberries is to not wash them after removing the caps. However, it is difficult for consumers to distinguish which insecticide is on the strawberries. Thus, these results suggest that soaking strawberries in a 3% vinegar or salt solution and rinsing with running water is an efficient way to remove insecticides from strawberries at home.

### 3.4. Dietary Exposure Risk Assessment

The EDIs (in mg/kg body weight/day) of the four analyzed insecticides were evaluated and are shown in Table 2. For different sexes, the median EDI values were higher for women: 0.49 × 10^−5^ for chlorfenapyr, 0.19 × 10^−4^ for cyenopyrafen, 0.75 × 10^−5^ for indoxacarb, and 0.17 × 10^−4^ for spirotetramat. Relatively low EDI values for chlorfenapyr (0.31 × 10^−5^), cyenopyrafen (0.10 × 10^−4^), indoxacarb (0.48 × 10^−5^), and spirotetramat (0.11 × 10^−4^) were observed in men. This observation can be explained by women having a higher daily consumption of strawberries (5.86 g/d) than men (4.66 g/d) and women having a lower average body weight (58.3 kg) than men (73.3 kg) (Table S6).

Additionally, the chronic risks posed by contaminants were evaluated using the risk quotient (RQ) calculated from the estimated dietary intake (DI) and the reported acceptable daily intake (ADI) for the analyzed insecticides (Table 2). An RQ value exceeding 1 signifies that pesticides pose an unacceptable risk to humans, whereas an RQ value below 1 indicates minimal risk [43]. The estimated RQ values for men and women consuming strawberries were all below 1, indicating minimal risk to human health.

## 4. Conclusions

Dissipation patterns of the four typical insecticides on strawberries with or without caps were observed over a period of 14 days, and the insecticide half-lives were 4.43–6.36 days for chlorfenapyr, 6.86–11.2 days for cyenopyrafen, 8.04–14.9 days for indoxacarb, and 11.4–20.4 days for spirotetramat. Remarkably, all four insecticides were distributed mostly in strawberry caps compared to the fruits, which suggests that removing the strawberry caps could remove most of the analyzed insecticides (61.3–80.8%). Moreover, the removal efficiencies of chlorfenapyr, cyenopyrafen, indoxacarb, and spirotetramat were evaluated using two different household washing methods and four washing solutions. The results suggest that soaking strawberries in a 3% vinegar or 3% salt solution and rinsing them with running water is the recommended way to remove these four insecticides from strawberries during household washing. Furthermore, for chlorfenapyr, 2–4 times lower residue levels were observed after removing caps without washing than in other washing processes, indicating that an efficient way to remove chlorfenapyr from strawberries is to remove the caps. This paper reports that some household washing solutions can effectively reduce insecticide residues on strawberries and ensure that humans have a healthy diet. Additionally, it could be also readily adapted to other soft fruits or other similar produce, not only strawberries.

## Figures and Tables

**Table 1:** The half-life and other statistical parameters for target insecticides in strawberries.

		Chlorfenapyr	Cyenopyrafen	Indoxacarb	Spirotetramat
With Caps	Determination coefficient (R^2^)	0.962	0.931	0.934	0.889
	k (day^−1^)	0.109	0.0617	0.0465	0.0340
	Half-life (days)	6.35	11.2	14.9	20.4
Without Caps	Determination coefficient (R^2^)	0.971	0.926	0.888	0.985
	k (day^−1^)	0.156	0.101	0.0862	0.0606
	Half-life (days)	4.43	6.86	8.04	11.4

**Table 2:** Median and worst estimate daily intakes (EDI) and risk quotients (RQ) of four insecticides for South Koreans of different genders.

		Chlorfenapyr		Cyenopyrafen		Indoxacarb		Spirotetramat	
		EDI	RQ	EDI	RQ	EDI	RQ	EDI	RQ
Male	Median	0.31 × 10^−5^	0.12 × 10^−3^	0.10 × 10^−4^	0.20 × 10^−3^	0.48 × 10^−5^	0.48 × 10^−3^	0.11 × 10^−4^	0.21 × 10^−3^
	Worst	0.44 × 10^−5^	0.17 × 10^−3^	0.13 × 10^−4^	0.26 × 10^−3^	0.53 × 10^−5^	0.53 × 10^−3^	0.22 × 10^−4^	0.44 × 10^−3^
Female	Median	0.49 × 10^−5^	0.19 × 10^−3^	0.16 × 10^−4^	0.31 × 10^−3^	0.75 × 10^−5^	0.75 × 10^−3^	0.17 × 10^−4^	0.34 × 10^−3^
	Worst	0.69 × 10^−5^	0.27 × 10^−3^	0.21 × 10^−4^	0.40 × 10^−3^	0.84 × 10^−5^	0.84 × 10^−3^	0.35 × 10^−4^	0.69 × 10^−3^
ADI (mg/kg body weight/day)		0.026		0.051		0.010		0.050	

## Data Availability

The data of the present study are available from the corresponding author upon request.

## Supplementary Materials

The following supporting information can be downloaded at: https://www.mdpi.com/article/10.3390/foods12061248/s1, Figure S1: Field trial locations conducted in South Korea; Table S1: Information of four insecticides spayed onto strawberry; Table S2: Detailed instrument conditions of HPLC-MS/MS for cyenopyrafen, indoxacarb, spirotetramat, and metabolites; Table S3: Detailed instrument conditions of GC-MS/MS for chlorfenapyr; Table S4: Storage stabilities (%) for different store periods (days) at deep-frozen temperature (below −20 °C) observed for target insecticides in strawberries; Table S5: Residual concentrations of pesticides in strawberries with or without caps; Table S6: Daily strawberry consumption rate and average body weight of different genders and age groups.

## References

[1] Afrin S., Gasparrini M., Forbes-Hernandez T.Y., Reboredo-Rodriguez P., Mezzetti B., Varela-López A., Giampieri F., Battino M. (2016). Promising Health Benefits of the Strawberry: A Focus on Clinical Studies. J. Agric. Food Chem..

[2] Lozowicka B., Jankowska M., Hrynko I., Kaczynski P. (2015). Removal of 16 Pesticide Residues from Strawberries by Washing with Tap and Ozone Water, Ultrasonic Cleaning and Boiling. Environ. Monit. Assess.

[3] Song L., Zhong Z., Han Y., Zheng Q., Qin Y., Wu Q., He X., Pan C. (2020). Dissipation of Sixteen Pesticide Residues from Various Applications of Commercial Formulations on Strawberry and Their Risk Assessment under Greenhouse Conditions. Ecotoxicol. Environ. Saf..

[4] Verdenelli R.A., Lamarque A.L., Meriles J.M. (2012). Short-Term Effects of Combined Iprodione and Vermicompost Applications on Soil Microbial Community Structure. Sci. Total Environ..

[5] Raghavendra K., Barik T.K., Sharma P., Bhatt R.M., Srivastava H.C., Sreehari U., Dash A.P. (2011). Chlorfenapyr: A New Insecticide with Novel Mode of Action Can Control Pyrethroid Resistant Malaria Vectors. Malar. J..

[6] Salazar-López N.-J., Aldana-Madrid M.-L., Silveira-Gramont M.-I., Aguiar J.-L. (2016). Spirotetramat—An Alternative for the Control of Parasitic Sucking Insects and Its Fate in the Environment.

[7] Wang Z., Cang T., Wu S., Wang X., Qi P., Wang X., Zhao X. (2018). Screening for Suitable Chemical Acaricides against Two-Spotted Spider Mites, Tetranychus Urticae, on Greenhouse Strawberries in China. Ecotoxicol. Environ. Saf..

[8] Bajwa U., Sandhu K.S. (2014). Effect of Handling and Processing on Pesticide Residues in Food—A Review. J. Food Sci. Technol..

[9] Acoglu B., Omeroglu P.Y. (2021). Effectiveness of Different Type of Washing Agents on Reduction of Pesticide Residues in Orange (*Citrus sinensis*). LWT.

[10] Harinathareddy A., Prasad N.B.L., Devi K.L., Raveendranath D., Ramesh B. (2015). Risk Mitigation Methods on the Removal of Pesticide Residues in Grapes Fruits for Food Safety. Res. J. Pharm. Biol. Chem. Sci..

[11] Polat B., Tiryaki O. (2020). Assessing Washing Methods for Reduction of Pesticide Residues in Capia Pepper with LC-MS/MS. J. Environ. Sci. Health Part B.

[12] Raveendranath D., Murthy K.S.R., Vijayalakshmi B., Reddy A.H., Ramesh B. (2014). Residual Behavior of Chlorpyriphos on Cultivated Gherkin and Its Removal Using Various Household Risk Mitigation Methods. Res. J. Pharm. Biol. Chem. Sci..

[13] Soliman K.M. (2001). Changes in Concentration of Pesticide Residues in Potatoes during Washing and Home Preparation. Food Chem. Toxicol..

[14] Yang T., Doherty J., Zhao B., Kinchla A.J., Clark J.M., He L. (2017). Effectiveness of Commercial and Homemade Washing Agents in Removing Pesticide Residues on and in Apples. J. Agric. Food Chem..

[15] Hanafi A., Elsheshetawy H.E., Faied S.F. (2016). Reduction of Pesticides Residues on Okra Fruits by Different Processing Treatments. J. Verbr. Lebensm..

[16] JEONG D.-K., LEE H.-J., BAE J.-Y., JANG Y.-S., HONG S.-M., KIM J.-H. (2019). Chlorfenapyr Residue in Sweet Persimmon from Farm to Table. J. Food Prot..

[17] Li Y., Xu J., Zhao X., He H., Zhang C., Zhang Z. (2021). The Dissipation Behavior, Household Processing Factor and Risk Assessment for Cyenopyrafen Residues in Strawberry and Mandarin Fruits. Food Chem..

[18] Liu Y., Su X., Jian Q., Chen W., Sun D., Gong L., Jiang L., Jiao B. (2016). Behaviour of Spirotetramat Residues and Its Four Metabolites in Citrus Marmalade during Home Processing. Food Addit. Contam. Part A.

[19] Liu Y., Li S., Ni Z., Qu M., Zhong D., Ye C., Tang F. (2016). Pesticides in Persimmons, Jujubes and Soil from China: Residue Levels, Risk Assessment and Relationship between Fruits and Soils. Sci. Total Environ..

[20] Food and Agriculture Organization of the United Nations, World Health Organization (1993). Pesticide Residues in Food, 1992 Evaluations: Part I Residues.

[21] Codex Recommended Methods of Sampling for the Determination of Pesticide Residues for Compliance with MRLs. https://www.iaea.org/resources/manual/codex-recommended-methods-of-sampling-for-the-determination-of-pesticide-residues-for-compliance-with-mrls.

[22] OECD (2021). Test No. 509: Crop Field Trial.

[23] Islam M.A., Amin S.M.N., Brown C.L., Juraimi A.S., Uddin M.K., Arshad A. (2022). Determination of the Most Efficient Household Technique for the Reduction of Pesticide Residues from Raw Fish Muscles. Foods.

[24] Noh H.H., Shin H.W., Kim D.J., Lee J.W., Jo S.H., Kim D., Kyung K.S. (2021). Effect of Processing on Residual Buprofezin Levels in Ginseng Products. Int. J. Environ. Res. Public Health.

[25] EURL|Residues of Pesticides|Analytical Quality Control and Method Validation Procedures for Pesticide Residues Analysis in Food and Feed. https://www.eurl-pesticides.eu/docs/public/tmplt_article.asp?CntID=727.

[26] Prevalence of Undernourishment 2019–2021 (Percent). https://www.fao.org/interactive/hunger-map-light/en/.

[27] Pesticide Fact Sheets. http://npic.orst.edu/npicfact.htm.

[28] Patra S., Ganguly P., Barik S.R., Samanta A. (2018). Dissipation Kinetics and Risk Assessment of Chlorfenapyr on Tomato and Cabbage. Environ. Monit. Assess.

[29] Xu F., Xu D., Hu M., Chen L., Xu C., Zha X. (2022). Dissipation Behaviour, Residue Analysis, and Dietary Safety Evaluation of Chlorfenapyr on Various Vegetables in China. Food Addit. Contam. Part A.

[30] Kabir M.H., El-Aty A.M.A., Kim S.-W., Rahman M.M., Chung H.S., Lee H.S., Shin H.-C., Shim J.-H. (2017). Decline Pattern and Risk Assessment of Cyenopyrafen in Different Varieties of Asian Pear Using Liquid Chromatography and Tandem Mass Spectrometry. Food Sci. Biotechnol..

[31] Lee C.-R., Hong J.-H., Lim J.-S., Lee K.-S. (2011). Residue Patterns of Azoxystrobin and Cyenopyrafen in Grape between Rainshield and Plastic House Conditions. Korean J. Pestic. Sci..

[32] Kaur H., Sharma S., Kang B.K. (2021). Estimation of Indoxacarb and Thiamethoxam Residues in Chilli. Int. J. Environ. Anal. Chem..

[33] Mohapatra S., Siddamallaiah L., Matadha N.Y., Udupi V.R., Raj D.P., Gadigeppa S. (2019). Dissipation of Neonicotinoid Insecticides Imidacloprid, Indoxacarb and Thiamethoxam on Pomegranate (*Punica granatum* L.). Ecotoxicol. Environ. Saf..

[34] Saimandir J., Gopal M. (2009). Application of Indoxacarb for Managing Shoot and Fruit Borer of Eggplant (*Solanum melongena* L.) and Its Decontamination. J. Environ. Sci. Health Part B.

[35] Sakthiselvi T., Paramasivam M., Vasanthi D., Bhuvaneswari K. (2020). Persistence, Dietary and Ecological Risk Assessment of Indoxacarb Residue in/on Tomato and Soil Using GC–MS. Food Chem..

[36] Szpyrka E., Matyaszek A., Słowik-Borowiec M. (2017). Dissipation of Chlorantraniliprole, Chlorpyrifos-Methyl and Indoxacarb—Insecticides Used to Control Codling Moth (*Cydia pomonella* L.) and Leafrollers (*Tortricidae*) in Apples for Production of Baby Food. Environ. Sci. Pollut. Res..

[37] Chahil G.S., Mandal K., Sahoo S.K., Singh B. (2014). Risk Assessment of Mixture Formulation of Spirotetramat and Imidacloprid in Chilli Fruits. Environ. Monit. Assess.

[38] Mohapatra S., Kumar S., Prakash G.S. (2015). Residue Evaluation of Imidacloprid, Spirotetramat, and Spirotetramat-Enol in/on Grapes (*Vitis vinifera* L.) and Soil. Environ. Monit. Assess.

[39] Mohapatra S., Deepa M., Lekha S., Nethravathi B., Radhika B., Gourishanker S. (2012). Residue Dynamics of Spirotetramat and Imidacloprid in/on Mango and Soil. Bull Enviro. N Contam. Toxicol..

[40] Wu W.-z., Li J.-y., He J., Chen Q.-b., Shan Z.-j. (2016). Degradation Dynamics of Spirotetramat Residue in Citrus. J. Ecol. Rural Environ..

[41] Saber A.N., Malhat F.M., Badawy H.M.A., Barakat D.A. (2016). Dissipation Dynamic, Residue Distribution and Processing Factor of Hexythiazox in Strawberry Fruits under Open Field Condition. Food Chem..

[42] Ravindranath D., Sri Rama Murthy K., Vijayalakshmi B., Reddy A., Prasad D. (2014). Persistence of Profenophos and Quinolphos on Cultivated Cucumber and Its Removal. Int. J. Pharmacogn. Phytochem. Res..

[43] Liu C., Lu D., Wang Y., Huang J., Wan K., Wang F. (2014). Residue and Risk Assessment of Pyridaben in Cabbage. Food Chem..

