# Supplementary material for: Dissipation of Four Typical Insecticides on Strawberries and Effects of Different Household Washing Methods
Source: Foods. 2023 Mar 15;12(6):1248. doi: 10.3390/foods12061248 (PMC10048472; doi:10.3390/foods12061248)
Supplement: Supplementary file 1 [file foods-12-01248-s001.zip › foods-2281461-supplementary.pdf]

Article

Dissipation of Four Typical Insecticides on Strawberries and Effects of Different Household Washing Methods

Wenting Wang, Jong-Wook Song, Seong-Hoon Jeong, Jung-Hoon Jung, Jong-Su Seo and Jong-Hwan Kim *

Environmental Safety-Assessment Center, Korea Institute of Toxicology (KIT), Jinju 52834, Republic of Korea
* Correspondence: jjong@kitox.re.kr

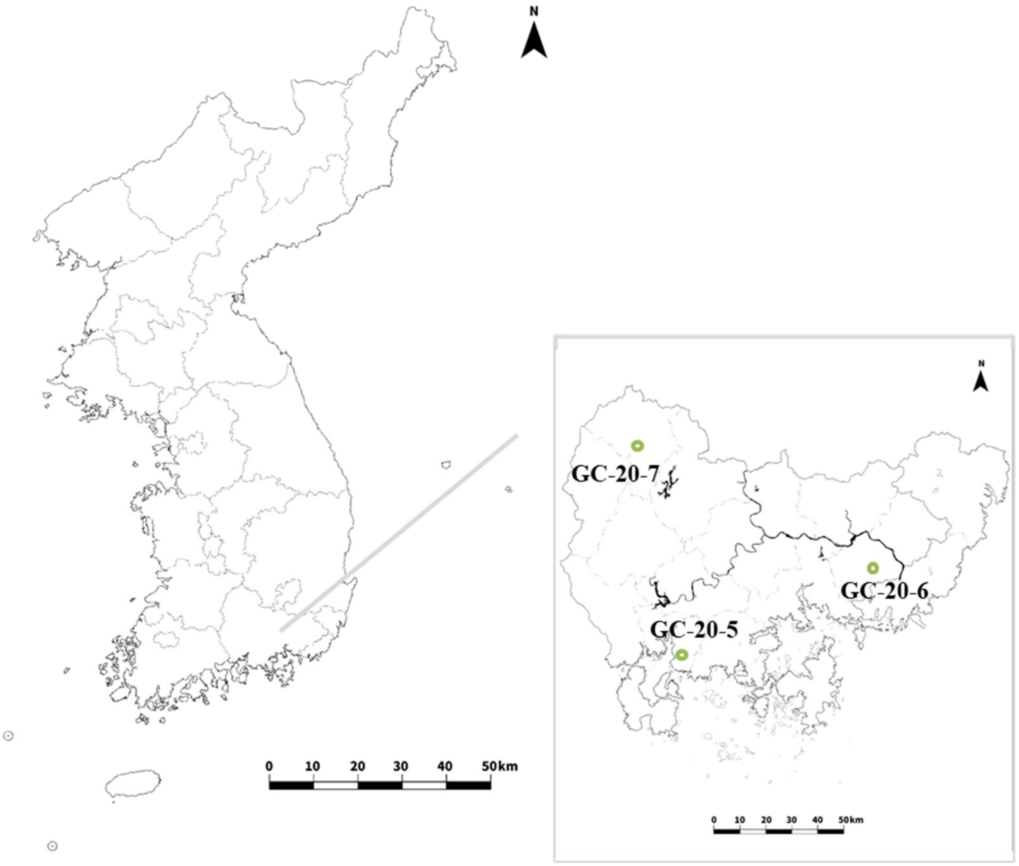

Figure S1 Field trial locations conducted in South Korea (NGII, 2019)

Table S1 Information of four insecticides spayed onto strawberry

Pesticides	Formulation Type	Dilution Rate	Application Rate		Application times	Internal (days)
			Application Solution (L/10 a)	Active Ingredient (kg/10 a)		
**Chlorfenapyr**	Suspension concentrate	2,000	167–175	0.0067–0.007	2	7
**Cyenopyrafen**	Suspension concentrate	2,000	163–185	0.0204–0.0231	3	7
**Indoxacarb**	Suspension concentrate	1,000	164–175	0.0082–0.0088	3	7
**Spirotetramat**	Suspension concentrate	2,000	160–175	0.0018–0.0019	3	7

Table S2 Detailed instrument condition of HPLC-MS/MS for cyenopyrafen, indoxacarb, spirotetramat and metabolites

Parameter	Conditions
**System**	Agilent 1260/6460 QQQMSD system
**Column**	Agilent Poroshell 120 EC-C18 (2.1 mm × 100 mm × 2.7 μm)
**Mobile phase**	5 mM ammonium formate, 0.1% formic acid in water:<br>5 mM ammonium formate, 0.1% formic acid in methanol = 20:80 (v/v)
**Flow rate**	200 mL/min
**Injection volume**	2 μL
**Column temp.**	40°C
**Ionization mode**	Positive ion electrospray
**MRM mode**	(see below)
**Gas temp.**	300°C
**Gas flow**	10 L/min

MRM mode:

Compounds	Precursor ion (m/z)	Product ions (m/z), (collision energy, (V)) Quantification	Product ions (m/z), (collision energy, (V)) Qualification	Fragmentor voltage (V)
Cyenopyrafen	394.25	310.2 (24)	254.2 (32)	155
Indoxacarb	528.1	150.0 (19)	218.2 (21)	110
Spirotetramat	374.2	330.2 (10)	302.2 (10)	90
BYI08330-cis-keto-hydroxy	318.1	300.1 (10)	268.3 (20)	90
BYI08330-mono-hydroxy	304.2	254.1 (18)	119.0 (40)	135
BYI08330 enol-glucoside	464.2	302.3 (20)	216.1 (46)	105
BYI08330-*cis*-enol	302.1	270.2 (18)	216.1 (22)	135

Table S3 Detailed instrument condition of GC-MS/MS for chlorfenapyr

Parameter	Conditions		
**System**	GC-Triple Quad MS, Bruker, USA		
**Column**	RESTEK RXi®-5sil MS (30 m × 0.25mm × 0.25 µm)		
**Injector temp.**	260℃		
**Injection volume**	1 µL (Splitless)		
**Oven**	180℃ (2 min)-20℃/min-320℃ (Hold for 3 minutes)		
**Carrier Gas**	Helium (1.2 mL/min)		
**Ionization mode**	EI mode		
**MRM mode**	**Precursor ion (m/z)**	**Product ion (m/z), (Collision energy, (V))**	
		**Quantification**	**Qualification**
	247.0	200.0 (25)	227.0 (15)
**Electron energy**	-70 eV		
**Transfer line temp.**	250℃		
**Source temp.**	200℃		

Table S4 Storage stabilities (%) for different store period (days) at deep-frozen temperature (below -20°C) observed for target insecticides in strawberry

Field trial site ID	Fortification level (mg/kg)	Stability (%) Store period (days)							
		**Chlorfenapyr**	**Cyenopyrafen**	**Indoxacarb**	**Spirotetramat**	**BYI08330-*cis*-keto-hydroxy**	**BYI08330-mono-hydroxy**	**BYI08330 enol-glucoside**	**BYI08330-*cis*-enol**
SC-20-5	0.5	104 (278)	100 (60)	100 (64)	77.1	90.2	92.0	103	93.2
					(299)				
GH-20-6		87.0 (262)	114 (47)	104 (59)	86.7	87.3	98.0	108	94.9
					(284)				
GC-20-7		102 (243)	92.5 (27)	102 (39)	88.1	85.7	95.9	108	93.5
					(268)				

Table S5 Residual concentrations of insecticides in strawberry with or without caps

Types	Day after last application	Mean Residue ± SD (n=3, mg/kg)								
		Chlorfenapyr	Cyenopyrafen	Indoxacarb	Spirotetramat	BYI08330-cis-keto-hydroxy	BYI08330-mono-hydroxy	BYI08330-enol-glucoside	BYI08330-cis-enol	∑Spirotetramat
With Caps	0	0.27 ± 0.097	0.99 ± 0.069	0.53 ± 0.14	0.74 ± 0.083	0.050 ± 0.030	<LOQ	0.023 ± 0.0078	0.38 ± 0.23	1.3 ± 0.38
	1	0.24 ± 0.048	0.98 ± 0.079	0.49 ± 0.057	0.70 ± 0.096	0.048 ± 0.029	<LOQ	0.022 ± 0.0082	0.37 ± 0.24	1.2 ± 0.41
	2/3	0.20 ± 0.074	0.83 ± 0.098	0.48 ± 0.037	0.63 ± 0.065	0.058 ± 0.039	<LOQ	0.023 ± 0.0054	0.29 ± 0.19	1.1 ± 0.28
	5	0.15 ± 0.047	0.65 ± 0.087	0.37 ± 0.032	0.51 ± 0.059	0.054 ± 0.036	<LOQ	0.023 ± 0.0051	0.30 ± 0.20	0.96 ± 0.29
	7	0.11 ± 0.033	0.61 ± 0.10	0.35 ± 0.019	0.53 ± 0.058	0.058 ± 0.046	<LOQ	0.028 ± 0.0039	0.30 ± 0.15	0.99 ± 0.23
	14	0.081 ± 0.014	0.48 ± 0.14	0.30 ± 0.045	0.43 ± 0.11	0.080 ± 0.058	<LOQ	0.030 ± 0.0001	0.20 ± 0.16	0.79 ± 0.23
Without Caps	0	0.071 ± 0.056	0.28 ± 0.097	0.12 ± 0.031	0.58 ± 0.33	<LOQ	<LOQ	<LOQ	<LOQ	0.60 ± 0.33
	1	0.061 ± 0.034	0.23 ± 0.097	0.11 ± 0.030	0.54 ± 0.31	<LOQ	<LOQ	<LOQ	<LOQ	0.54 ± 0.31
	2/3	0.044 ± 0.023	0.17 ± 0.075	0.077 ± 0.023	0.49 ± 0.28	<LOQ	<LOQ	<LOQ	<LOQ	0.49 ± 0.28
	5	0.036 ± 0.022	0.15 ± 0.060	0.070 ± 0.031	0.44 ± 0.28	<LOQ	<LOQ	<LOQ	<LOQ	0.44 ± 0.28
	7	0.020 ± 0.012	0.14 ± 0.050	0.060 ± 0.013	0.35 ± 0.21	<LOQ	<LOQ	<LOQ	<LOQ	0.35 ± 0.21
	14	0.011 ± 0.0081	0.064 ± 0.016	0.042 ± 0.013	0.25 ± 0.20	<LOQ	<LOQ	<LOQ	<LOQ	0.25 ± 0.20

Table S6 Daily strawberry consumption rate and average body weight of different gender and age

Gender	Daily consumption rate (g/d)	Average body weight (kg)
Male	4.66	73.3
Female	5.86	58.3
